# Emerging roles of piRNAs in cancer: challenges and prospects

**DOI:** 10.18632/aging.102417

**Published:** 2019-11-13

**Authors:** Ye Cheng, Qian Wang, Wei Jiang, Yonghua Bian, Yang zhou, Anxing Gou, Wenling Zhang, Kai Fu, Weihong Shi

**Affiliations:** 1 Jiangsu Research Center for Primary Health Development and General Practice Education, Jiangsu Vocational College of Medicine, Yancheng, China; 2 Department of General Surgery, Nanjing First Hospital, Nanjing Medical University, Nanjing, Jiangsu, China; 3 Department of Gastroenterology, Nanjing First Hospital, Nanjing Medical University, Nanjing, Jiangsu, China

**Keywords:** piRNA, PIWI proteins, cancer, transposon silencing

## Abstract

PiRNAs are a small class of non-coding small RNAs newly discovered in recent years. Millions of piRNAs have been discovered to date, and more than 20,000 piRNA genes have been found in the human genome. Due to the relatively small number of studies related to piRNA, our understanding of piRNAs is very limited. Currently, the clear biological function of piRNAs is transposon mobilization inhibition by promoting transcript degradation and regulating chromatin formation. In addition, piRNAs can form piRNA-PIWI protein complexes with some members of the PIWI branch of the Argonaute protein. Based on these biological functions, piRNAs and PIWI proteins are important in maintaining the genomic integrity of germline cells. Because of this, the popularity of piRNAs research has been focused on its role in germline cells for a long time after the discovery of piRNAs. As the field of research expands, there is growing evidence that piRNAs and PIWI proteins are abnormally expressed in various types of cancers, which may be potential cancer biomarkers and cancer therapeutic targets. In this review, we will focus on the relationship between piRNAs and PIWI proteins and cancers based on previous research, as well as their significance in cancer detection, grading and treatment.

## INTRODUCTION

Previous studies have shown that more than 90% of the human genome may be transcribed. However, only about 2% of the genes are translated after transcription. In other words, only about 2% of the human genome has protein-coding functions, and the rest of the gene transcriptome is non-coding RNAs (ncRNAs), which can be divided intot wo major categories of housekeeping ncRNA and regulation ncRNA [1, 2]. Depending on their molecular size, all regulated ncRNAs can be further divided into small ncRNAs (less than 200 nucleotides) and large ncRNAs (greater than 200 nucleotides). Large ncRNAs are mainly long non-coding RNAs (lncRNAs) and circular RNAs (circRNAs), and small ncRNAs are diverse, including circRNAs, microRNAs (miRNAs) and transfer RNAs (tRNAs), ribosomal RNAs (rRNAs), small nucleolar RNAs (snoRNAs), short interfering RNAs (siRNAs) and PIWI interacting RNAs (piRNAs) [3, 4]. A large number of studies have shown that circRNAs, miRNAs and lncRNAs play essential roles in various diseases including cancer.

The P-element-induced wimpy testis (PIWI)-interacting RNAs (piRNAs) are the newest member of the ncRNA family. The length of the piRNAs is 26~30nt, which is close to the length of the miRNAs. By searching the relevant databases, we can see that about 23,000 piRNA genes were found in the human genome, close to the number of proteins encoding the mRNA gene (about 20,000), but far more than the number of miRNA genes (about 2,000) [5, 6]. The huge number of genes indicates that piRNAs may be involved in gene regulation, but their specific mechanism remains to be studied.

In 2001, Aravin, et al. found that the repetitively related small interfering RNAs extracted from the repetitive genomic elements can inhibit the duplication of the D-sequence encoding genes, thus the existence of piRNAs was first demonstrated, but they did not figure out what this small interfering RNA is [7]. By 2006, biologists have been able to isolate and purify piRNA from other small ncRNAs, but still cannot explain their biological functions. Subsequently, more and more studies have shown that these new small ncRNA often binding to the PIWI subfamily of Argonaute proteins and function in mammalian germ cells through this mechanism [8–10].

The PIWI protein was first discovered in Drosophila, and was revealed to be involved in the maintenance and self-renewal of germline stem cells in Drosophila [11, 12]. There are three Argonaute proteins belonging to the PIWI protein subfamily in germline cells, Aub, Piwi and AGO3 [13, 14]. The piRNAs have a unique structure of 2'-O-methyl at the 3' end, and the PIWI proteins are capable of specifically binding to this structure [15–17]. PIWI is a nuclear protein and involved in the silencing of retrotransposons and the control of male germ line mobility. In addition, PIWI is also involved in the occurrence of sperm [18]. Knockout mutations in PIWI proteins may result in defects in sperm development [19]. Therefore, PIWI proteins have been extensively studied in germline and stem cells. In this review, we will discuss current understanding of piRNAs biogenesis, the functional and molecular mechanisms of piRNAs and PIWI proteins. In addition, we will discuss the potential applications of piRNAs and PIWI proteins as disease biomarkers for cancer diagnosis and treatment.

## PIRNA biogenesis

Depending on the sources, piRNAs can be divided into three groups: lncRNA-derived piRNAs, mRNA-derived piRNAs, and transposon-derived piRNAs. Transposon-derived piRNAs are transcribed from two genomic strands, thus producing both piRNAs and antisense piRNAs; mrna-derived piRNAs are usually derived from 3' untranslated regions (UTRs) and are processed and processed. Treatment; lncRNA-derived piRNAs are produced from the entire transcript [20, 21]. Only transposon-derived piRNAs were studied more thoroughly among the three sources of piRNAs.

**Figure 1:**
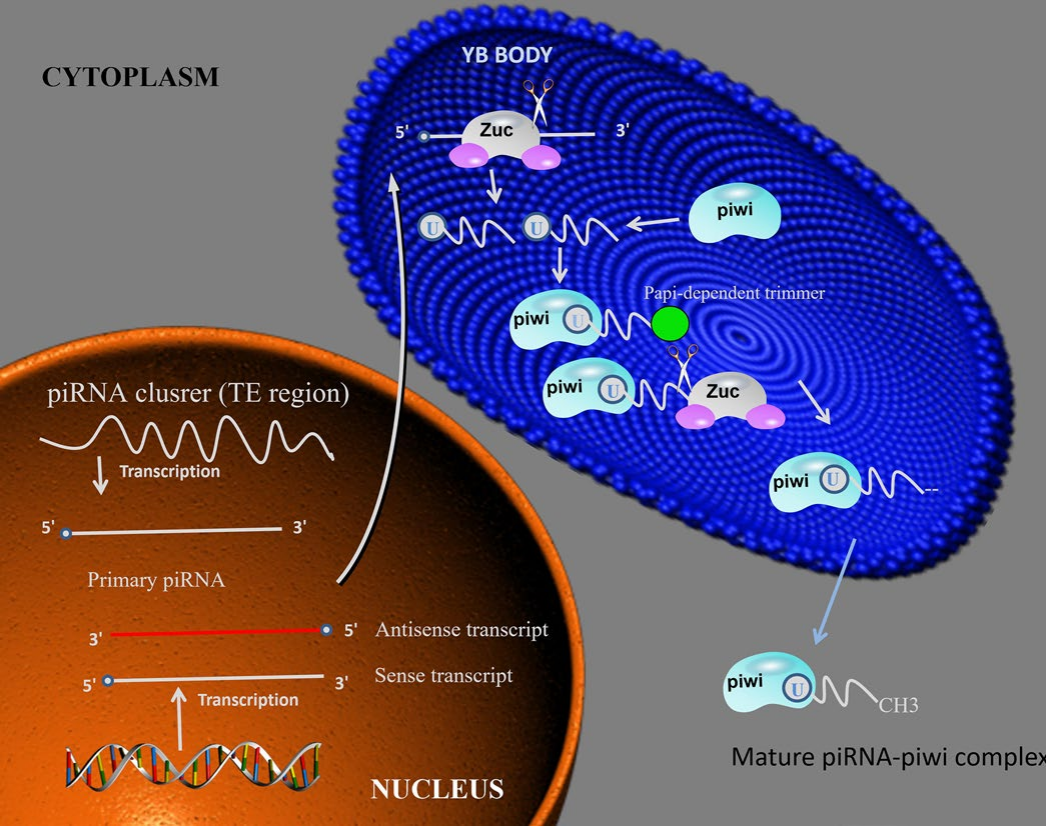
**piRNA biosynthesis mechanism.** piRNA intermediates form a complex with piwi, then the complex is cleavage by Zuc or Papi-dependent trimmer to form 3’end. After the complex methylation in cytoplasm, the mature piRNA-piwi complex is producted. Abbreviations: Zuc: zucchini.

**Figure 2:**
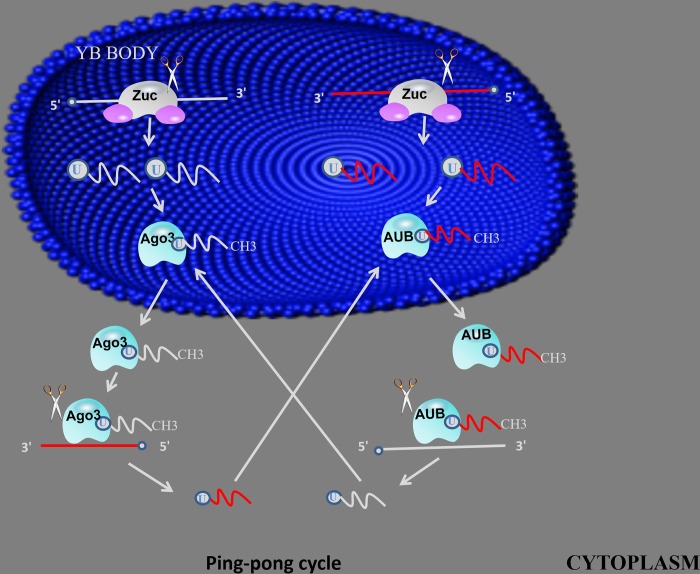
**ping-pong mechanism.** primary piRNAs bind to AGO3 or AUB proteins to form piRNA/Ago3 or piRNA/AUB complexes, generate new piRNAs using piRNA and piRNA/Ago3 or piRNA/AUB complexes as templates, using new generated piRNA to synthesis another piRNA in same method.

Mature piRNAs are 26-30 nt in length and are close to the length of miRNA (20-24 nt) and siRNA (21-25 nt). In terms of precursors, piRNAs are mostly transcribed to be large (up to 200 kb), independent of Dicer ribonuclease-treated single-stranded precursors. In contrast, for miRNAs and siRNAs, RNaseIII Dicer is used to form stem loops or double-stranded precursor [22–24]. These precursors are usually generated by specific genomic locations containing repeating elements, a process usually done by a pathway independent of the dicer. In addition, newborn piRNAs require post-transcriptional modification to become mature piRNAs. The biogenesis of piRNAs involves two major pathways: primary amplification pathway and a secondary amplification pathway - also often referred to as the ping-pong mechanism (Figure 1 and Figure 2) [21].

### Primary amplification

piRNAs are derived from piRNA clusters, which are mostly composed of various transposon DNA elements, suggesting that piRNAs may be antisense relative to retrotransposon-derived RNA [8, 9]. Primary synthesis relies on RNA polymerase II in the nucleus to transcribe small nucleotide sequences from the piRNA gene cluster to form long single-stranded precursor piRNA, which is then transferred to the cytoplasm. In Drosophila glandular cells, the initial transcription is catalytic cleavage by the endonuclease Zucchini. The piRNA precursor fragment produced after cleavage is integrated into the PIWI protein and excised to the final length by 3' to 5' exo-cleavage, and then separately bound to the PIWI protein to form a piRNA/PIWI protein complex [25].

After the piRNA/PIWI protein complex is formed, it migrates back to the nucleus to reach the target gene, and through the complementary base pairing of piRNA and DNA, activates the silencing mechanism and blocks the transcription of the target gene. Thus, piRNAs are transcriptional regulators acting on transposable elements and by the recruitment of histone methyltransferases, resulting in the establishment of heterochromatin for transcriptional silencing [26].

### Secondary amplification

After the primary piRNA produced by the primary synthesis is transferred from the nucleus to the cytoplasm, a second amplification by the ping-pong mechanism is required [25]. Primary piRNAs bind to AGO3 or AUB proteins to form piRNA/Ago3 or piRNA/AUB complexes, and piRNA/Ago3 complexes can be used as templates to generate new RNAs as substrates for new piRNA formation, and the resulting new piRNAs can Load Aub protein. The RNA produced by the piRNA/Aub protein complex as a template is then used as a substrate by a similar process to form a new piRNA/Ago3 complex. This interaction with a product of one piRNA molecule as a substrate for the synthesis of another piRNA molecule to achieve simultaneous amplification of the two molecules is ping-pong amplification [27]. The researchers found ping-pong mechanisms in some primitive animals such as zebrafish, black-bellied porpoises and sponges, suggesting that ping-pong mechanisms are important in the early stages of biological evolution [28]. However, subsequent studies have shown that the biogenesis of piRNAs does not depend on the ping-pong mechanism during mouse adult spermatogenesis. There is more evidence that in mammals, the piRNA/PIWI mechanism exhibits diversity and requires more in-depth exploration.

## PiRNA function

Previous studies about piRNAs have been focused on transcriptional and post-transcriptional levels, only few studies proceed to the function of piRNAs at the post-translational level. However, translation and post-translational modification are essential for tumor biogenesis and development, so we need more in-depth and specific research on the translation and post-translational modification of piRNAs in the future.

**Figure 3:**
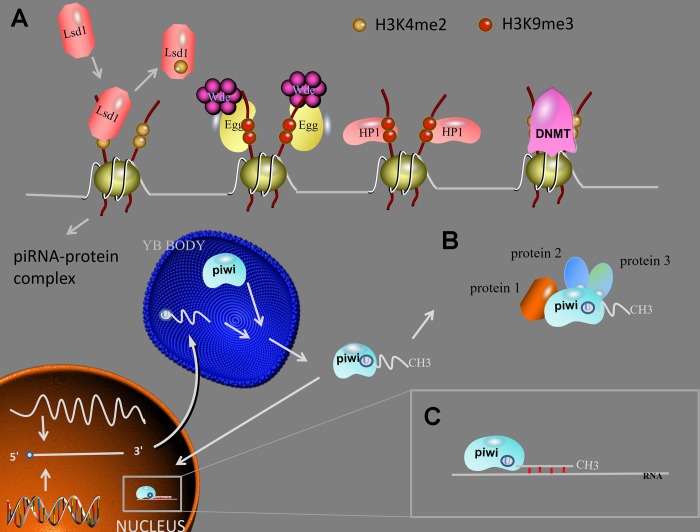
**piRNA function.** (**A**) Transposon silencing. At TGS level, Lsd1 removes activating H3K4me2 marks from promoter regions, Egg and Wde H3K9me3 marks to the target DNA, HP1 lead to heterochromatin formation, DNMT methylate genic CpG sites. After the mature piRNA-piwi complex is formed in the cytoplasm. (**B**) piRNAs/piwi complex-protein interaction. The interaction between piRNAs/piwi and proteins alter the subcellular localization of proteins and facilitate the interaction of multiple proteins. (**C**) At PTGS level, the piRNAs/piwi complex bind to targeted RNAs and impede their function by sequence complementary. Abbreviations: TGS: transcription gene silencing; PTGS: post-transcription gene silencing; H3K9me3: histone 3 lysine 9 trimethylation; H3K4me2: histone 3 lysine 4 dimethylation; Lsd1: Lysine-specific demethylase1; Egg: Eggless; Wde: Windei; HP1: heterochromatin protein 1; DNMT: DNA methyltransferase.

In different species, the sequence and function of piRNAs are very different, so it is difficult to establish a complete system to explain the function of piRNAs, and only a certain degree of summary of the discovered functions [29]. According to the existing research evidence, piRNAs play an important role in transposon silencing, gene and protein regulation (Figure 3).

### Transposon silencing

Through building an RNA-induced silencing complex (RISC), piRNAs have function in RNA silencing. RISC can bind to PIWI proteins, and direct the PIWI proteins to the transposon targets [30]. Transposable elements are a class in many post-live objects. The discovered mobile genetic factors can be “jumped” from one location of the genome to another through a series of processes such as cutting and re-integration. Because transposable elements can bring new genetic material to the genome, in some cases, they can start or shut down certain genes like a switch, and often cause DNA rearrangements such as deletion, duplication or inversion of the genome. It is closely related to biological evolution and may be related to individual development and cell differentiation. Transposable elements (TEs) can be grouped into two types according to their mode of replication: 1) retrotransposons, which are transcribed into RNA intermediates; 2) DNA transposons, which do not need transcription to be mobilized [31]. TEs can lead to genetic diversity and instability, are potentially highly pathogenic, and can further affect the pathogenic process through chromosomal rearrangements, deleterious mutations, and gene deregulation [32–35].

Silencing of the transposon resulting from DNA methylation can lead to silencing of the transcriptional gene [36]. Previous studies have shown that PIWI proteins are associated with methylation of repetitive elements and maintenance of transposon suppression [13, 37]. The TE sequence is used as a silencing target by PIWI, and transcriptional repression is closely related to the presence of a transposon or its residues in introns or close genes [38–39]. Thus, decreased expression of piRNA clusters may result in increased TE activity [40]. Therefore, a complete idea can be established that PIWI translocates to the nucleus via piRNA, interacting with the nascent transcript or DNA of the target site, resulting in heterochromatin formation and transcriptional repression.

### Transcriptional gene silencing (TGS)

After the mature piRNA-piwi complex is formed in the cytoplasm, the complex enters the nucleus and combines with the genomic target to form a new complex. When the new complex combined with Panoramix (Panx), the complex will recruit silencing components, and TGS will begin. First, Lysine-specific demethylase1 (Lsd1) removes activating histone 3 lysine 4 dimethylation (H3K4me2) marks from promoter regions, this result will lead to the inhibit of RNA Pol II transcription [41]. Then, Eggless (Egg) and its co-factor Windei (Wde) add repressive histone 3 lysine 9 trimethylation (H3K9me3) marks to the target DNA. Subsequently, the silencing component been recruited is heterochromatin protein 1 (HP1), which will lead to heterochromatin formation. In addition, piRNA/piwi complex also recruits DNA methyltransferase (DNMT) to methylate genic CpG sites (TE protein-coding), altering transcriptional activity [42].

In Aplysia neurons, as Rajasethupathy P, et al. concluded that piRNA induced CREB2 promoter methylation [43]. Fu A, et al. demonstrated that the over-expression of piR- 021285 facilitated ARHGAP11A methylation at a CpG site within the 5 UTR/first exon, decreased the expression of mRNA, which promote apoptosis and then, inhibiting breast cancer cell apoptosis [96]. Yan H, et al. discovered that piRNA-823 recruited DNA methyltransferases DNMT3A and DNMT3B, then increased global DNA methylation and inhibiting tumor suppressor p16INK4A expression in primary CD138+ multiple myeloma cells [90].

### Post-transcriptional gene silencing (PTGS)

After the discovery of TGS, the researchers find that piRNAs inhibit the function of target through regulate post-transcriptional networks, which is similar to miRNA made, scilicet piRNA-RNA interactions. These RNAs interacting with piRNA include lncRNA [44], mRNA [92] and pseudogenes [53]. PiRNA interaction requires the 5’-end of piRNA occur base pairing, which arise strict base pairing within 2-11 nt and less stringent base pairing within 12-21 nt [45]. In addition, piRNA-piwi complex can promote RNA repression through recruit carbon catabolite-repressed 4- negative on TATA-less (CCR4 NOT) and Smaug (Smg) to build a specific pi-RISC, which can arise imperfect base-pairing with RNA [46, 47].

For instance, Peng L, et al. propose that piR-55490 can bind to the 3’-UTR of mTOR, thereby lead to degradation of mRNA and lung cancer development suppression [93]. Liu X, et al. demonstrate that piR-30188 can inhibit OIP5-AS1 expression by bind to lncRNA OIP5-AS1, thereby suppressing glioma cell through the miR-367/CEBPA/TRAF4 pathway [44].

In addition, piRNA-piwi ribonucleoprotein complex can lead to TEs post-transcriptionally silence, thus maintains genome integrity [48], this result can drive and promote genome evolution and must be tightly regulated because of their over-activity is harm for the host [49]. In Ping-Pong piRNA amplification, Krimper can recruite symmetric dimethyl-arginine (sDMA)-modified mature ribonucleoprotein complexes. Besides that, Krimper also can interact with unloaded Ago3, thus bringing these together. Since complex and Ago3 both have a piwi domain with RNase H endonuclease activity, the complex newly established can selectively detect and cleave transposon RNA, thus making TEs to be silenced at post-transcriptional level [50].

### Gene and protein regulation

PiRNAs can act as a regulator of genes and proteins. Related mechanisms have been mentioned in the part of transposon silencing. For example, piR-39980 can suppress RRM2 through extensive sequence complementary binding to the 3'UTR of RRM2 [51]. PiR-1245 can bind to a panel of target genes including ATF3, BTG1, DUSP1, FAS, NFKBIA, UPP1, SESN2, TP53, INP1 and MDX1, and then, build up specific piRNA silencing complexes (pi-RISC), hence, leading to RNA suppression via abnormal base-pairing [52]. SEPW1P is a retroprocessed pseudogene of SEPW1. PiRNA-36712 can compete with RNAs produced by SEPW1P (SEPW1P RNA) for miR-7 and miR-324, thus effect SEPW1 mRNA, finally inhibits SEPW1 expression. It's worth mentioning that up-regulation of SEPW1 caused by down-regulation of piR-36712 will inhibit P53 [53].

PiRNAs also regulate its interacting proteins stability by binding to it. For instance, piRNA-54265 can bind to PIWIL2 protein and promoting for the formation of PIWIL2/STAT3/phosphorylated-SRC (p-SRC) complex, which activates STAT3 signaling and promotes proliferation, metastasis and chemoresistance of CRC cells [54]. Lee YJ, et al discovered two tumor suppressor proteins, SERPINA1 and LRAT, which were directly regulated as endogenous piR-36026 target genes in breast cancer cells. This discovery makes piR-36026 available for molecular therapy in breast cancer [55].

## PiRNAs and PIWI proteins in cancer

**Figure 4:**
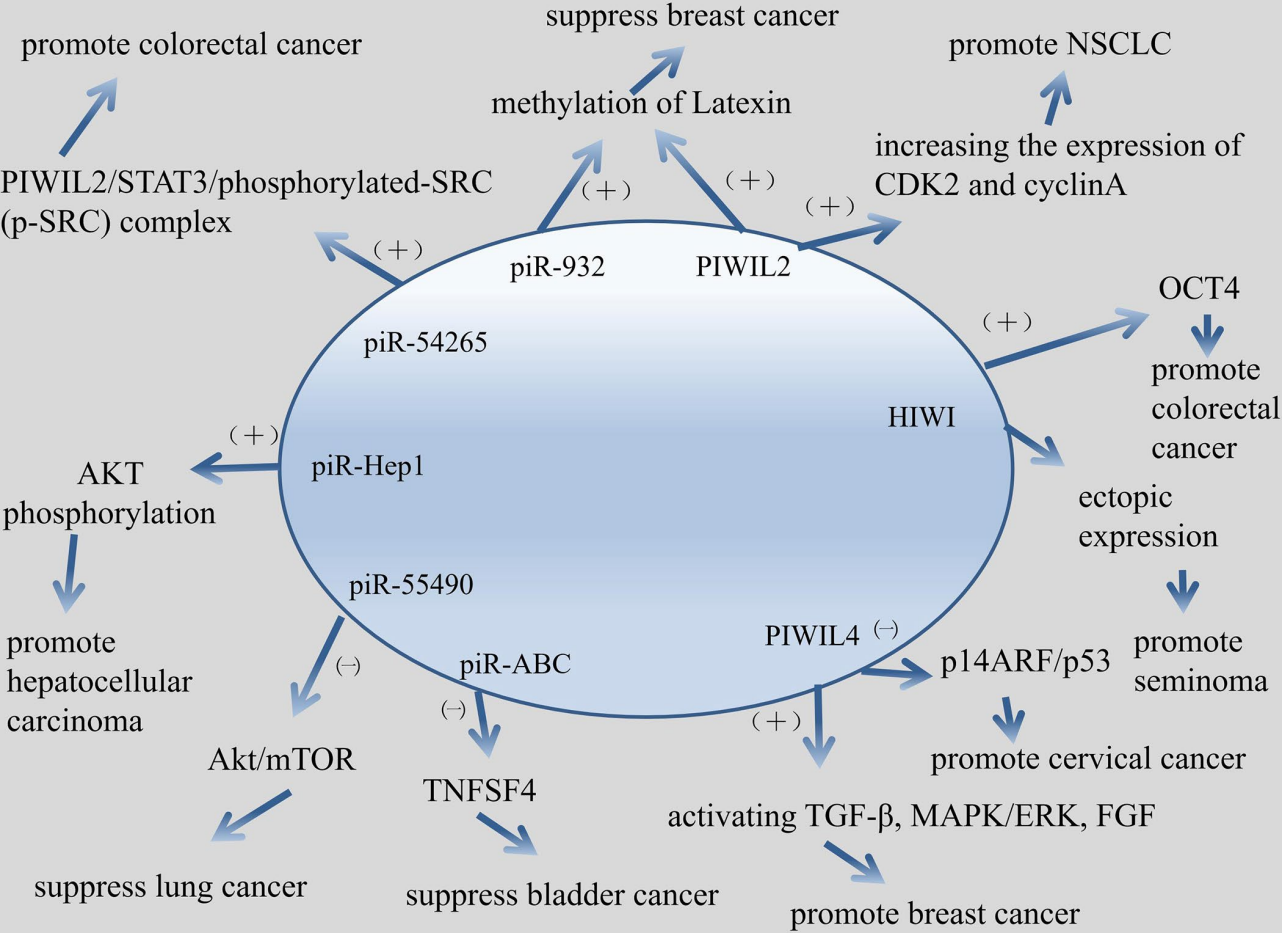
**Mechanism about piRNA/PIWI protein involved in cancer in this article.**

From germ cells to stem cells to cancer cells, with the deepening of piRNAs research, people are increasingly thinking about whether piRNAs and PIWI proteins play a role in human diseases, especially in cancer. We summarized the mechanisms about the involvement of piRNA or PIWI protein in this article (Figure 4). It is not difficult to find out through the relevant data that germ cells, stem cells and cancer cells have some key biological characteristics, such as the ability to rapidly proliferate and self-renew. It has been mentioned in the foregoing that the piRNAs pathway maintains germline stem cells by regulating the self-renewal mechanism of germ cells. Then there is also a self-renewal mechanism similar to germ cells in rapidly dividing cancer cells. In this regard, a number of recent studies have revealed a previously unrecognized association between piRNAs and PIWI proteins and human cancer (Supplementary Table 1). Next we will discuss and summarize the role of the piRNAs pathway in the development and progression of cancer and the role of piRNAs and PIWI proteins as markers and therapeutic targets for cancer diagnosis and prognosis.

### PIWI proteins and cancer

In humans, there are four PIWI proteins, which are PIWI-like protein 1 (PIWIL1, or HIWI), PIWIL2 (HILI), PIWIL3 (HIWI3), and PIWIL4 (HIWI2) [77]. PIWI proteins belongs to the argonaute family, Piwi subfamily. The feature domain of PIWI proteins mainly position on Q9VKM1(538 - 829).

As the function of PIWI proteins, firstly, PIWI proteins mediates the repression of transposable elements during meiosis by forming complexes composed of piRNAs and PIWI proteins and governs the methylation and subsequent repression of transposons, thus generating transposon silencing. Transposon silencing and many accompanying phenomena are associated with inhibition and promotion of many types of cancer [25, 56, 57]. Then, PIWI proteins mediates a somatic signaling mechanism required for the maintenance of germline stem cells to produce and maintain a daughter germline stem cell [11, 12, 58, 59].

PIWI proteins play a role in invasion, migration, proliferation, division and survival of cancer. Knockout of PIWIL1 gene leads to altered expression of some genes, such as ABL1, ABL2, DOCK2, ZNF503, PDE4 and so on, which encoded proteins are involved in cellular invasion and migration [60]. PIWIL1 can induce epithelial-mesenchymal transition and confer endometrial cancer cells with stem-like properties, such as ability of migration and invasion [61]. Knockdown of PIWIL2 in glioma cells can inhibits glioma cell migration [62]. PIWIL2 is up-regulated in NSCLC cells, and PIWI2 promotes cell proliferation by increasing the expression of CDK2 and cyclinA [63]. PIWIL4 promotes cell division, migration and survival of breast cancer by activating TGF-β, MAPK/ERK, and FGF signaling pathways [64].

Based on previous studies, there is a strong correlation between the expression of PIWI protein and poor clinical prognosis, so these year's research on PIWI protein and tumorigenesis is very active. Previous studies have shown that high expression of PIWI protein and esophageal squamous cell carcinoma, gastric cancer, liver cancer, cholangiocarcinoma, intestinal cancer, breast cancer, non-small cell lung cancer, renal cell carcinoma, bladder cancer, ovarian cancer, melanoma. The aggressiveness of sarcomas, gliomas, and leukemia is associated with poor clinical outcomes.

Just after the existence of piRNAs was confirmed, some researchers suggested that PIWI protein was associated with varieties of tumors. Qiao D, et al. first proposed that HIWI protein was related to seminoma because of the essential role HIWI play in germ cell proliferation [65]. Later studies have shown that PIWI proteins were related with gastric cancer. Liu X, et al suggested that HIWI protein may function in the gastric cancer development and can be a potential target for cancer therapy [66]. Wang Y, et al. demonstrated that the PIWI subfamily protein was a key molecular factor in the tumor biogenesis and development. PIWI protein could act as a potential biomarker for gastric cancer prognosis evaluation [67]. PIWI protein could be used as a carcinogen and biomarker for breast cancer. Wang DW, et al. concluded that HIWI protein function as oncogenic role in breast cancer [68]. Zhang H, et al. suggested that PIWIL2 could be the potential targets for blocking the metastasis of breast cancer through promoting the methylation of Latexin [69]. Krishnan P, et al. proposed that PIWI protein in dysregulated piRNAs pathway have been identified to act as a novel markers for breast cancer prognostication [70]. The PIWI protein can also be found in the study of glioma. Wang X, et al. claimed that the reduction of HIWI inhibited tumor growth in vivo, and HIWI acted as an oncogene to take part in glioma progression [71]. Sun G, et al. suggested that Hiwi may be a key factor in glioma progression and could be used as a potential molecular marker for malignant gliomas in pathological diagnosis and prognosis evaluation [72].

PIWI proteins appear frequently in related studies of HCC. Xie Y, et al. concluded that HIWI may play an essential role in the progression of hepatocellular carcinoma and may be the target for cancer therapy [73]. Zhao YM, et al. demonstrated that PIWI may play a key role in HCC proliferation and metastasis, thus could be a potential prognostic factor for HCC, especially in well-differentiated type [74]. Zeng G, et al. indicated that the molecular chaperone PIWIL2/PIWIL4 had potential to be a molecular marker for prognosis judgment for HCC [75]. Certainly, PIWI protein participant in germline tumors. Su C, et al. proposed that PIWIL4 may play a carcinogenic role in cervical cancer through the p14ARF/p53 pathway and may serve as a new therapeutic target for the future [76]. There are also many articles about the relationship between PIWI protein and colorectal cancer. Li D, et al. claimed that PIWIL2 involved in colon cancer via regulation of matrix metallopeptidase 9 transcriptional activity [78]. Liu C, et al. indicated that the invasiveness of tumor could be assessed by measuring the level of HIWI in colorectal cancer [79]. Litwin M, et al. concluded that the expression of HIWI mRNA level, which related to the expression of OCT4, was completely higher in colorectal cancer tissues. Therefore, HIWI may play a role in the pathogenesis of colorectal cancer [80]. Oh SJ, et al. demonstrated that PIWIL2-positive cells play a positive role in the progression of colorectal cancer [81].

### PiRNA and cancer

Unlike mirna, most piRNAs are not complementary to potential target gene mRNAs, suggesting that piRNAs may be involved in epigenetic regulation rather than post-transcriptional regulation, controlling a variety of biological phenomena including cancer [82, 83]. Epigenetic global changes in cancer include DNA hypomethylation, histone hypoacetylation, and gene-specific DNA hypermethylation, leading to oncogene activation (R-ras, cyclin D2) [84, 85], and tumor suppressor silencing (RB1, p16) [86]. In cancer tissues, aberrantly expressed piRNAs implicated by global hypomethylation and local hypermethylation may be potential cancer-specific features [87, 88]. There is increasing evidence that although only a small number of piRNAs are currently expressed in somatic tissues, several piRNAs have been involved in the development of cancer. In conclusion, piRNAs are involved in the proliferation, apoptosis, metastasis, and invasion of cancer cells, and may be potential prognostic and diagnostic biomarkers in the development of cancer. Specific examples are followed.

Chu H, et al. suggested that piRABC (DQ594040) could affected the expression of TNFSF4 protein and played an important role in the development of bladder cancer [89]. Yan H, et al. proposed that piRNA-823 was capable of promoting angiogenesis and played a carcinogenesis role in multiple myeloma (MM), thus providing a possibility for the development of piRNA-targeted therapeutic strategies in MM [90]. Jacobs DI, et al. Revealed that piR-8041 can reduce cell proliferation, induce cell cycle arrest and apoptosis, and inhibit cell survival pathways. Thus, piR-8041 may be a potential therapy target for glioblastoma [91]. In recent years, researches about piRNAs in lung cancer has made great progress. Peng L, et al. indicated that piR-55490 inhibited the growth of lung carcinoma by suppressing the activation of Akt/mTOR pathway [92]. Zhang SJ, et al. concluded that piR-651 could inhibit cell proliferation, migration, invasion, as well as induced apoptosis, and then regulated NSCLC oncogenic activity. Therefore, piR-651 could be a potential diagnosis marker of NSCLC [93]. Mei Y, et al. demonstrated that piRNA/piRNA-L could interact with proteins in pathophysiological and physiological conditions, therefore, piRNA/piRNA-L might act as a regulatory role in NSCLC [94]. PiRNAs have been found to play an important role in the development, progression and any other aspects of breast cancer. Hashim A, et al. Suggested that piRNAs, which show a specific expression pattern in breast tumors, targeted key cancer cell pathways [95]. Fu A, et al. proposed that piR-21285 functioned in the development of breast cancer through the correlative epigenetic mechanism [96]. Huang G, et al. claimed that piR-4987, piR-20365, piR-20485 and piR-20582 have been shown to be up-regulated in breast cancer and might serve as biomarkers for breast cancer [97]. Tan L, et al. revealed that piRNA-36712 was a novel tumor suppressor and may act as a promising predictor for the prognosis of breast cancer [43]. Lee YJ, et al. Indicated that piR-36026 played a role in the control of tumor suppressor genes, and mediated breast cancer progression in vivo and in vitro.46 PiRNA was also associated with urinary tumors. Li Y, et al. concluded that the up-regulation of piR-32051 piR-39894 piR-43607 were highly associated with clear cell renal cell carcinoma (ccRCC) metastasis, late clinical stage and poor cancer-specific survival [98]. Iliev R, et al. Demonstrated that the urinary piR-823 detection helps RCC diagnosis [99]. Busch J, et al. suggested that based on the aberrant expression of piR-57125, piR-30924, piR-38756 in ccRCC tissues, these piRNAs could be used as a potential prognostic biomarker for ccRCC [100].

Tumors in the digestive system are very common, hence, there are many studies about piRNA in this field. Cui L, et al. proposed that piR-651, piR-823 might be valuable biomarkers for detecting circulating gastric cancer cells [101]. Cheng J, et al. revealed that piR-823 plays a crucial role in inhibiting the development and progression of gastric cancer [102]. Cheng J, et al. claimed that piR-651 might be involved in the progression of gastric cancer, and was a potential marker for gastric cancer diagnosis [103]. Martinez VD, et al. indicated that piR-59056, piR-32105, piR-58099 could be tumor markers in gastric cancer, furthermore, could effectively stratified GC patients into low and high-risk of recurrence groups [104]. Law PT, et al. concluded that piR-Hep1 was upregulated in HCC. Silencing of piR-Hep1 inhibited cell dynamic and invasiveness, and could lead to a decrease in the level of active AKT phosphorylation [105]. Chu H, et al. Demonstrated that piR-15551 might be generated from LNC00964-3, which might be involved in the biogenesis and development of CRC [106]. Weng W, et al. suggested that piR-1245 target a group of tumor suppressor genes include ATF3, BTG1, DUSP1, FAS, NFKBIA, UPP1, SESN2, TP53INP1 and MDX1, hence, piR-1245 play a carcinogenic role and may serve as potential prognostic biomarker in colorectal cancer [52]. Vychytilova-Faltejskova P, et al. proposed that piR-5937, piR-28876 could serve as potential biomarkers for early detection of colon cancer [107]. Mai D, et al. revealed that piRNA-54265 play an oncogenic function and might be a therapeutic target in CRC by promoting the formation of PIWIL2/STAT3/phosphorylated-SRC (p-SRC) complex, which activates STAT3 signaling and promotes proliferation, metastasis and chemoresistance of CRC cells [54].

In addition, there are also many studies about piRNAs for some uncommon tumors. Das B, et al. claimed that piR-39980 possess very strong anti-tumor effect, and hence, has great potential for the treatment of fibrosarcoma [51]. Saad MA, et al. indicated that disorder of piRNAs (piR-35373, piR-266308, piR-58510 and piR-38034) caused by alcohol consumption might be involved in the pathogenesis of alcohol-related HNSCC [108].

## CONCLUSION AND FUTURE PERSPECTIVES

It has been more than ten years since the first discovery of piRNAs. The current understanding of piRNAs and PIWI protein is not clear enough. It is impossible to construct a complete knowledge network to explain the biogenesis and function of piRNAs and PIWI protein and their interaction. With the continuous development of high-throughput sequencing technology and bioinformatics, the gene regulation function of piRNAs has been paid more and more attention and has been gradually discovered. In recent years, research about piRNAs as tumor biomarker has become a hot topic. Similar to circRNA, many common assays can be used for piRNA study. For example, using High-throughput Sequencing (HTS) to assay new and known piRNAs; using Reverse Transcription-quantitative PCR (RT-qPCR) to assay exact piRNA copy number per cell and the relative expression; using RNA binding protein immunoprecipitation (RIP) to assay The interaction of piRNA-proteins; using Luciferase reporter system to assay the interaction of piRNA-target RNA.

At the same time, more and more studies have shown that the dysregulation of piRNAs is related to cancer, but the specific molecular mechanism leading to dysregulation of piRNAs in the development of cancer is still unclear, and further research is needed. In addition, there are many problems with cancer-related piRNAs studies. For example, piRNAs and PIWI proteins independently affect the proliferation, metastasis, invasion, and apoptosis of cancer cells, or whether piRNAs and PIWI protein act together on cancer cells. It is still unclear, so there is still a long time to study piRNAs in cancer. The way to go. But it is foreseeable that with the continuous efforts of scientists and the emergence of more and more new technologies, there will be more new ideas for studying the relationship between piRNAs and cancer in the near future, and research on piRNAs can actually treat cancer.

## Supplementary Material

Supplementary Table 1
