# Supplementary material for: Emerging roles of piRNAs in cancer: challenges and prospects
Source: Aging (Albany NY). 2019 Nov 13;11(21):9932–46. doi: 10.18632/aging.102417 (PMC6874451; doi:10.18632/aging.102417)
Supplement: Supplementary Table 1 [file aging-11-102417-s001.docx]

**Supplementary Table 1．A summary of cancer-related piRNAs and PIWI proteins.**

| **Cancer Type**  **and Symbol** | **Expression**  **in Tumors** | **Clinical value** | **Related Mechanisms** | PMID |
| --- | --- | --- | --- | --- |
| **Breast** **cancer** |  |  |  |  |
| piR-4987 piR-20365 piR-20485 piR-20582 | up | tumor markers | piR-4987, piR-20365, piR-20485 and piR-20582 have been shown to be up-regulated in breast cancer and might serve as biomarkers for breast cancer. | 23229900 |
| piR-34736 piR-36249 piR-35407  piR-36318  piR-34377 | down | tumor markers | Exert transcriptional and post-transcriptional gene regulatory actions. | 25313140 |
| piR-36026 | up | tumor promoter | piR-36026 played a role in the control of tumor suppressor genes, and mediated breast cancer progression in vivo and in vitro. | 27289065 |
| piR-932  piwil2 | up | tumor promoter | the combination of piR-932 and PIWIL2 could be the potential target for blocking the metastasis of breast cancer through promoting the methylation of Latexin. | 23992744 |
| piR-36712 | down | tumor suppressor | piRNA-36,712 was a novel tumor suppressor and may act as a [promising](D:/%E6%9C%89%E9%81%93%E8%AF%8D%E5%85%B8/Dict/8.5.2.0/resultui/html/index.html#/javascript:;) predictor for the prognosis of breast cancer. | 30636640 |
| piR-21285 | [variation](D:/%E6%9C%89%E9%81%93%E8%AF%8D%E5%85%B8/Dict/8.5.3.0/resultui/html/index.html#/javascript:;) | mediated tumorigenesis | piR-21285 functioned in the development of breast cancer through the correlative epigenetic mechanism. | 26210741 |
| hiwi | up | tumor promoter | HIWI protein function as oncogenic role in breast cancer. | 25292027 |
| Piwil3  Piwil4 | Piwil3-up  Piwil4-down | tumor marker | PIWI protein in dysregulated piRNA pathway have been identified to act as a novel marker for breast cancer prognostication. | 27177224 |
| **Colorectal cancer** |  |  |  |  |
| piR-1245 | up | tumor promoter | piR-1245 acts as an oncogene and promotes tumor progression. | 29382334 |
| piR-15551 | up | tumor promoter | piR-15551 might be generated from LNC00964-3, which might be involved in the biogenesis and development of CRC. | 25740697 |
| piR-5937 piR-28876 | up | tumor markers | piR-5937, piR-28876 could serve as potential biomarkers for early detection of colon cancer. | 29976566 |
| piRNA-54265 | up | tumor promoter | piRNA-54265 play an oncogenic function and might be a therapeutic target in CRC by promoting the formation of PIWIL2/STAT3/phosphorylated-SRC (p-SRC) complex, which activates STAT3 signaling and promotes proliferation, metastasis and chemoresistance of CRC cells. | 30555542 |
| hiwi | up | tumor markers | the invasiveness of tumor could be assessed by measuring the level of HIWI in colorectal cancer. | 22261620 |
| hiwi  hili | up | tumor promoter | the expression of HIWI mRNA level, which related to the expression of OCT4, was completely higher in colorectal cancer tissues. Therefore, HIWI may play a role in the pathogenesis of colorectal cancer. | 26026091 |
| piwil2 | up | tumor promoter | PIWIL2-positive cells play a [positive](D:/%E6%9C%89%E9%81%93%E8%AF%8D%E5%85%B8/Dict/8.5.2.0/resultui/html/index.html#/javascript:;) role in the progression of colorectal cancer. | 23110023 |
| piwil2 |  |  | Piwil2 involved in colon cancer via regulation of matrix metallopeptidase 9 transcriptional activity. | 23104504 |
| **Gastric** **cancer** |  |  |  |  |
| piR-823 | down | tumor suppressor | Increased expression of piR-823 had tumor suppressive effects （Tumor suppressive role） | 22047710 |
| piR-59056 piR-32105  piR-58099 | up | tumor markers | piR-59056, piR-32105, piR-58099 could be tumor markers in gastric cancer, furthermore, could effectively stratified GC patients into low and high-risk of recurrence groups. | 25779424 |
| piR-651  piR-823 | down | tumor markers | piR-651, piR-823 might be valuable biomarkers for detecting circulating gastric cancer cells. | 21704610 |
| piR-651 | up | tumor marker | piR-651 might be involved in the progression of gastric cancer, and was a potential marker for gastric cancer diagnosis. | 21616063 |
| hiwi | up | tumor marker | HIWI protein may function in the gastric cancer development and can be a potential target for cancer therapy. | 16287078 |
| piwi | up | tumor promoter | the PIWI subfamily protein was an key molecular factor in the tumor biogenesis and development. PIWI protein could act as a potential biomarker for gastric cancer prognosis evaluation. | 22670175 |
| **Glioblastoma** |  |  |  |  |
| piRNA-8041 | down | tumor suppressor | piR-8041 can reduce cell proliferation, induce cell cycle arrest and apoptosis, and inhibit cell survival pathways. | 30701019 |
| hiwi | up | tumor promoter | the reduction of HIWI inhibited tumor growth in vivo, and HIWI acted as an oncogene to take part in glioma progression. | 25269862 |
| hiwi | up | tumor marker | hiwi may be a key factor in glioma progression and could be used as a potential molecular marker for malignant gliomas in pathological diagnosis and prognosis evaluation. | 21138738 |
| **Hepatocellular carcinoma** |  |  |  |  |
| piR-Hep1 | up | tumor promoter | Silencing of piR-Hep1 inhibited cell dynamic and invasiveness, and could lead to a decrease in the level of active AKT phosphorylation. | 23376363 |
| hiwi | up | tumor promoter | Hiwi may play an essential role in the progression of hepatocellular carcinoma and may be the target for cancer therapy. | 25370791 |
| hiwi | up | tumor marker | HIWI may play a key role in HCC proliferation and metastasis, thus could be a potential prognostic factor for HCC, especially in well-differentiated type. | 21989785 |
| piwil2  piwil4 | nuclear co-expression | tumor marker | the molecular chaperone Piwil2/Piwil4 had potential to be a molecular marker for prognosis judgment for HCC. | 27894076 |
| **Lung cancer** |  |  |  |  |
| piR-L-163 | down | tumor suppressor | piR-L-163 directly binds to phosphorylated ERM (p-ERM)  and play a critical role in ERM activation | 26095918 |
| piR-55490 | down | tumor suppressor | piR-55490 inhibited the growth of lung carcinoma by suppressing the activation of Akt/mTOR pathway. | 26408181 |
| piR-651 | down | tumor suppressor | piR-651 could inhibit cell proliferation, migration, invasion, as well as induced apoptosis, and then regulated NSCLC oncogenic activity. Therefore, piR-651 could be a potential diagnosis marker of NSCLC. | 29399156 |
| **Multiple Myeloma** |  |  |  |  |
| piR-823 | up | tumor promoter | piR-823 contributed to tumorigenesis by regulating DNA methylation and angiogenesis | 24732595 |
| **Pancreatic cancer** |  |  |  |  |
| piR-17061 | down | - | - | 25910082 |
| **Renal Cell Carcinoma** |  |  |  |  |
| piR-32051  piR-39894  piR-43607 | up | tumor promoter | the up-regulation of piR-32051 piR-39894 piR-43607 were highly associated with clear cell renal cell carcinoma (ccRCC) metastasis, late clinical stage and poor cancer-specific survival. | 25998508 |
| piR-57125  piR-30924 piR-38756 | down | tumor Suppressor | based on the [aberrant](D:/%E6%9C%89%E9%81%93%E8%AF%8D%E5%85%B8/Dict/8.5.2.0/resultui/html/index.html#/javascript:;) expression of piR-57125, piR-30924, piR-38756 in ccRCC tissues, these piRNAs could be used as a potential prognostic biomarker for ccRCC. | 26071182 |
| piR-823 |  | tumor markers | the urinary piR-823 detection helps RCC diagnosis. | 27919963 |
| **Seminoma** |  |  |  |  |
| hiwi | ectopic expression | tumor causer | HIWI protein was related to seminoma because of the essential role hiwi play in germ cell proliferation. | 12037681 |
| **Cervical cancer** |  |  |  |  |
| piwil4 | up | tumor promoter | PIWIL4 may play a carcinogenic role in cervical cancer through the p14ARF / p53 pathway and may serve as a new therapeutic target for the future. | 22483988 |
| **Ovarian cancer** |  |  |  |  |
| piwil1 | down | tumor suppressor | Over-expression of PIWIL1 reduces invasiveness of ovarian cancer cell line SKOV3. | 24932571 |
| **Bladder cancer** |  |  |  |  |
| piRABC (DQ594040)  hiwi | piRABC-down  hiwi-down | piRABC-tumor suppressor  hiwi-tumor marker | piRABC (DQ594040) could affected the expression of TNFSF4 protein and played a important role in the development of bladder cancer. | 25305452 |
| **Fibrosarcoma** |  |  |  |  |
| piR-39980 | down | tumor suppressor | piR-39980 possess very strong anti-tumor effect, and hence, has great potential for the treatment of fibrosarcoma. | 30362638 |
| **HNSCC** |  |  |  |  |
| piR-35373  piR-266308  piR-58510  piR-38034  piwil4 | up | tumor promoter | disorder of piRNAs (piR-35373, piR-266308, piR-58510 and piR-38034) caused by alcohol consumption might be involved in the pathogenesis of alcohol-related HNSCC. | 30854037 |
